# *Porphyromonas gingivalis* downregulates the immune response of fibroblasts

**DOI:** 10.1186/1471-2180-13-155

**Published:** 2013-07-10

**Authors:** Eleonor Palm, Hazem Khalaf, Torbjörn Bengtsson

**Affiliations:** 1 Division of Clinical Medicine, School of Health and Medical Sciences, Örebro University, Örebro, Sweden

**Keywords:** *Porphyromonas gingivalis*, Fibroblasts, Chemokines, Cytokines

## Abstract

**Background:**

*Porphyromonas gingivalis* is a key pathogen in periodontitis, an inflammatory disease leading to destruction of bone and tooth-supporting tissue. *P. gingivalis* possesses a number of pathogenic properties to enhance growth and survival, including proteolytic gingipains*.* Accumulating data shows that gingipains are involved in the regulation of host inflammatory responses. The aim of this study was to determine if *P. gingivalis* infection modulates the inflammatory response of fibroblasts, including the release of chemokines and cytokines. Human gingival fibroblasts or primary dermal fibroblasts were pre-stimulated with tumor-necrosis factor-α (TNF- α) and cocultured with *P. gingivalis*. Gingipain inhibitors were used to explore the effect of gingipains. CXCL8 levels were determined with ELISA and the relative levels of various inflammatory mediators were determined by a cytokine assay.

**Results:**

TNF-α-triggered CXCL8 levels were completely abolished by viable *P. gingivalis*, whereas heat-killed *P. gingivalis* did not suppress CXCL8. Accumulation of CXCL8 was partially restored by an arginine-gingipain inhibitor. Furthermore, fibroblasts produced several inflammatory mediators, notably chemokines, all of which were suppressed by viable *P. gingivalis*.

**Conclusion:**

These findings provide evidence that fibroblast-derived inflammatory signals are modulated by heat-instable gingipains, whereby the bacteria can escape killing by the host immune system and promote its own growth and establishment. In addition, we show that fibroblasts are important mediators of inflammation in response to infection and thereby play a crucial role in determining the nature and magnitude of the invasion of immune cells.

## Background

The gram-negative, anaerobic bacterium *Porphyromonas gingivalis* is considered to be one of the key pathogens in periodontitis, an inflammatory polymicrobial condition leading to bone and tissue destruction and eventually tooth loss [1]. Increasing evidence associate periodontitis to systemic diseases [2] and for instance, *P. gingivalis* has been found in atherosclerotic plaques [3,4] as well as in non-healing ulcers (unpublished data). *P. gingivalis* possess a number of pathogenic properties to enhance growth and survival such as fimbriae, lipopolysaccharides and gingipains. The gingipains, which are grouped into lysine-specific (Kgp) and arginine-specifik (Rgp) gingipains due to their specificity for cleavage after lysyl and arginyl residues, respectively, are cysteine proteases that have been linked to the establishment and growth of *P. gingivalis*. The gingipains are, like the fimbriae, important for the bacterial invasion and colonization. They are reactive against an array of different proteins, *e.g.* proteins of the complement and kallikrein system, coagulation factors and cytokines. Of particular interest, accumulating data shows that gingipains are involved in the regulation of host inflammatory responses. *P. gingivalis* stimulates an innate immune response and induces expression of inflammatory mediators, but can at the same time downregulate the host response. In other words, *P. gingivalis* has evolved several mechanisms to evade host immune system by invasion of host cells and disrupting signalling pathways by cytokine and receptor degradation [1,5-7].

Periodontitis is a chronic inflammation with associated bone-resorption and tissue destruction. This degenerative process is mainly a consequence of the hosts attempt to eliminate the bacterial load rather than the bacteria themselves. As a consequence to bacterial encounter, the host cells synthesize and release mediators attracting inflammatory cells to the site of infection, which in turn contribute to the characteristic tissue and bone destruction by release of proteolytic enzymes, induction of osteoclast formation and apoptosis of cells [1]. One important chemokine that attracts neutrophils to the site of infection is CXCL8. CXCL8 is expressed and produced by different cell types, including fibroblasts, neutrophils, endothelial cells, keratinocytes, epithelial cells and lymphocytes [8].

Innate immunity defence against invading pathogens involves their sensing through highly conserved pattern recognition receptors (PRRs). These receptors, including toll like receptors (TLRs), are expressed by a variety of cells, both immune and none-immune cells. For instance, human gingival fibroblasts (HGFs) are likely to encounter microbial invasion at an early stage of periodontitis and interact with bacteria and bacterial products, and several studies report a role of HGFs in periodontal inflammation [9-11]. HGFs have been shown to express TLRs and, although there are some inconsistencies in which TLRs that are expressed, the importance of fibroblasts in innate immunity has started to be revealed. Mahanonda and colleagues reported that HGFs express functional TLR 2, 3, 4 and 5, and that ligand binding to these receptors lead to the secretion of CXCL8 [12]. Uehara *et al.* demonstrated that HGFs express TLR 1–9, and that stimulation of TLR 2/6, 3, 4, 7/8 and 9 caused production of several inflammatory mediators [13]. However, increasing data suggest that fibroblasts are heterogeneous. Fibroblasts from different anatomic sites, and even subpopulations of fibroblasts from the same site, display distinct differences in morphology, extracellular matrix production, migratory phenotype and cell surface antigens [14].

Recently, our group showed that *P. gingivalis* target T cell derived interleukin (IL) 2 at the protein level and suppresses activator protein 1, a mechanism by which *P. gingivalis* benefits its own establishment by altering adaptive immune responses [15]. The aim of the present study is to characterize the effects of *P. gingivalis* on primary human fibroblasts and their derived inflammatory responses, with the hypothesis that initial establishment of *P. gingivalis* infection modulates immunoregulatory mechanisms of fibroblasts.

## Methods

### Isolation and culture of fibroblasts

Primary human skin fibroblasts were isolated by explanting pieces of dermis obtained from elective abdominal or chest surgery from three young donors. The tissue was removed using standard surgical procedures. Approval from the local Ethical Committee at Örebro County Council, Sweden, (no. 2003/0101), and informed consent was obtained from each patient. Fibroblasts were propagated from dermal preparations pieces by the explant technique. In brief, small pieces (half-millimeter) of dermis were allowed to adhere to culture plastic for a few minutes followed by addition of culture medium (Dulbecco’s modified Eagle medium (DMEM) supplemented with 10% fetal bovine serum (FBS) and 1 mg/ml gentamicin (all from Invitrogen Ltd, Paisley, UK). Gingival fibroblasts (HGF-1, ATCC CRL-2014) were purchased from the American Type Collection (Manassas, VA, USA). The fibroblasts were cultured to confluence and removed from culture plastic surface by incubation in 0.25% trypsin and 1 mM EDTA (Invitrogen Ltd, Paisley, UK) at 37°C for 5 minutes. The cells were plated in tissue culture flasks in DMEM with 10% FBS. Fibroblasts were used at passages 3–10.

### Preparation of *P. gingivalis*

*P. gingivalis* ATCC 33277 (American Type Culture Collection, Manassas, VA, USA) was cultured in fastidious anaerobe broth (29.7 g/liter, pH 7.2) under anaerobic conditions (80% N_2_, 10% CO_2_, and 10% H_2_) at 37°C in an anaerobic chamber (Concept 400 Anaerobic Workstation; Ruskinn Technology Ltd., Leeds, United Kingdom). The bacteria were harvested by centrifugation, washed and resuspended in Krebs-Ringer glucose buffer (KRG) (120 mM NaCl, 4.9 mM KCl, 1.2 mM MgSO_4_, 1.7 mM KH_2_PO_4_, 8.3 mM Na_2_HPO_4_, and 10 mM glucose, pH 7.3). Heat-killed *P. gingivalis* was prepared by incubation at 70°C for 1 h. To ensure that the bacteria were killed, 10 μl of the heat-killed suspension was spread on a fastidious anaerobe agar plate and incubated at 37°C for five days.

### Coculture of *P. gingivalis* and fibroblasts

In 0.5 ml DMEM supplemented with 10% FBS, primary dermal fibroblasts from each subject or gingival fibroblasts were seeded with a density of 50,000 cells/well in a 24-wells plate (Sarstedt, Inc, Newton NC, USA). After 24 hours, the fibroblasts were washed twice with phosphate buffered saline (PBS) (Invitrogen, Paisley UK) and 0.5 ml serumfree DMEM was added. After 24 hour of starvation, the medium was replaced with DMEM supplemented with 1% FBS. The cells were thereafter treated with viable *P. gingivalis,* at a multiplicity of infection (MOI) of 1:1, 1:10, 1:100 or 1:1000, or heat-killed *P. gingivalis* (MOI:1000). The cocultures were incubated for 1, 6, or 24 hours in 37°C in 5% CO_2_.

CXCL8 accumulation was induced by pre-stimulating fibroblasts with tumor necrosis factor-α (TNF-α) (50 ng/ml) for 6 hours prior to infection with *P. gingivalis.* The fibroblasts were stimulated with the previously mentioned concentrations of viable or heat-killed bacteria, respectively, for 24 hours in 37°C in 5% CO_2_.

To evaluate the role of gingipains, *P. gingivalis* was incubated with the Arg-gingipain inhibitor leupeptin (Roche Diagnostics Corporation, Indiana, USA) or the Lys-gingipain inhibitor cathepsin B inhibitor II (Calbiochem, Biocompare, CA, USA), for 1 hour prior to fibroblast stimulation. After stimulation with viable and heat-killed *P. gingivalis*, and/or TNF-α, leupeptin as well as cathepsin B inhibitor II, for 1, 6 or 24 hours, the supernatants were collected and stored in aliquots at −80°C prior to immunoassays.

### FITC-labeling of *P. gingivalis*

*P. gingivalis* was washed three times with PBS by centrifugation at 12000 rpm for three minutes, whereby the bacteria were resuspended in buffered saline (0.05 M Na_2_C0_3_, 0.1 M NaCl, pH 9.3) containing 0.2 mg/ml fluorescein isothiocyanate isomer (FITC) (Sigma-Aldrich, St. Louis, MO, USA), and incubated in darkness at room-temperature for 45 minutes. The bacteria were washed in PBS prior to fibroblast infection.

### Fluorescence microscopy

For fluorescence microscopy, fibroblasts were seeded on coverslips in multiwell plates and incubated for 24 hours. The fibroblasts were stimulated with FITC-labeled *P. gingivalis* (MOI:100) for 6 hours. The cells were washed twice with PBS, fixed with 4% paraformaldehyde (PFA) for 30 min at room temperature and washed with PBS. F-actin was visualized by incubating the cells with 2 units Alexa Fluor® 594 phalloidin and 100 μg/ml lysophosphatidylcholine in darkness for 1 h at room temperature. The nucleus was counterstained with 1 μg/ml 4′,6-Diamidino-2-Phenylindole, Dihydrochloride (DAPI) for 2 min (all dyes obtained from Invitrogen Ltd, Paisley, UK).

### Determination of cytokine production

CXCL8 was measured by Human IL-8 ELISA MAX Deluxe Set (BioLegend San Diego, CA, USA) according to the manufacturer’s instructions. All samples were run in duplicates. For the parallel determination of the relative levels of cytokines and chemokines, Human Cytokine Array Panel A (R&D System, Inc, Abingdon, UK) was performed according the manufacturer’s instructions. Briefly, cell culture supernatants from representative experiments were mixed with a cocktail of biotinylated detection antibodies and the sample/antibody mixture was incubated with the array where capture antibodies were spotted in duplicate on a nitrocellulose membrane. Any formed cytokine/detection antibody complex was then bound by its immobilized capture antibody on the membrane. Detection was performed by adding Streptavidin-Horseradish Peroxidase and chemiluminescent detection reagents, and the signal produced was in proportion to the amount of cytokine bound. Chemiluminescence was detected in the same manner as a Western blot (ChemiDoc XRS System, Bio-Rad Laboratories, CA, USA). The array determined the relative levels of 36 different cytokines, chemokines and acute phase proteins (Table 1).

**Table 1:** Cytokines, chemokines and acute phase proteins that are detectable in the performed cytokine profiler assay

C5a	IL-4	IL-32α
CD40 ligand	IL-5	CXCL10
G-CSF	IL-6	CXCL11
GM-CSF	CXCL8	CCL2
CXCL1	IL-10	MIF
CCL1	IL-12 p70	CCL3
sICAM-1	IL-13	CCL4
IL-1α	IL-16	CCL5
IL-1β	IL-17	CXCL12
IFN-γ	IL-17E	Serpin E1
IL-1ra	IL-23	TNF-α
IL-2	IL-27	sREM-1

### Data analysis

CXCL8 experiments were performed in three independent experiments (one experiment/primary fibroblast strain) in duplicates to confirm the reproducibility of the results. Experiments with human gingival fibroblasts were performed in three independent experiments. Statistical analysis with Student’s *t*-test was performed using GraphPad Prism (GraphPad Software, La Jolla, CA, USA). All data are presented as mean values with standard deviation. A value of p < 0.05 was considered statistically significant. One experiment was performed for the cytokine array.

## Results

### P. gingivalis invades fibroblasts

**Figure 1:**
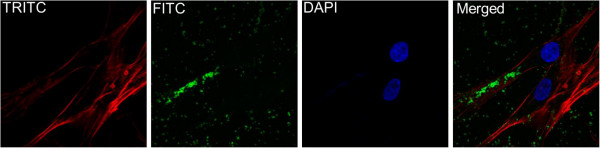
***P. gingivalis *adheres to and invades dermal fibroblasts.** Dermal fibroblasts were seeded on a coverslip and incubated for 24 h. The cells were then stimulated with FITC-labeled *P. gingivalis* (MOI:100) for 6 h. F-actin was visualized by incubating the cells with Alexa Fluor® 594 phalloidin (TRITC) and the nuclei were visualized by counterstaining the cells with DAPI. Magnification is 60× (Olympus FluoviewTM FV1000, Germany).

The morphology of fibroblasts following treatment with different concentrations of viable and heat-killed *P. gingivalis* was examined by light microscopy. No obvious morphological changes induced by the bacteria were observed (data not shown). The interaction between *P. gingivalis* and fibroblasts was visualized by fluorescence microscopy. We found that *P. gingivalis* after 6 h effectively adhered to and invaded the fibroblasts (Figure 1).

### *P. gingivalis* affects the level of CXCL8 in a dose- and time-dependent manner

**Figure 2:**
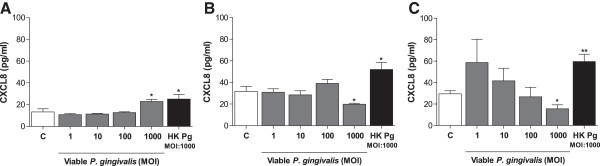
***P. gingivalis *suppresses basal level CXCL8 accumulation.** Primary dermal fibroblasts (50,000 cells/well) were stimulated with the indicated concentrations of viable or heat-killed *P. gingivalis* (HK Pg, MOI:1000) for 1 h **(A)**, 6 h **(B)** and 24 h **(C)**. CXCL8 expression was increased following short-term exposure (1 h), while long-term treatment (>6 h) suppressed CXCL8 accumulation. Heat-killed *P. gingivalis* treated fibroblasts resulted in elevated CXCL8 expression both after short- and long-term treatment. The asterisks indicate significant differences compared to the untreated negative control **(C)**. *- p < 0.05; **- p < 0.01 (Student’s *t*-test).

Primary fibroblasts (50,000 cells/well) were stimulated with different concentrations of viable *P. gingivalis,* as well as heat-killed *P. gingivalis,* for 1 h, 6 h or 24 h (Figure 2). The highest concentration (MOI:1000) of either viable or heat-killed *P. gingivalis* significantly increased CXCL8 expression after short-term exposure (1 h), whereas lower concentrations of viable *P. gingivalis* (MOI:1, MOI:10, MOI:100) did not change the CXCL8 level compared to the unstimulated control. However, long-term treatment (6 and 24 hours) with viable bacteria (MOI:1000) resulted in a significant reduction in CXCL8 levels. Although not consistently statistically significant for all concentrations of viable bacteria tested, there is a tendency for decreasing CXCL8 levels with increasing MOI. Heat-killed *P. gingivalis* (MOI:1000) resulted in elevated CXCL8 production both after short- and long-term exposure of fibroblasts.

### *P. gingivalis* is involved in the degradation of CXCL8 protein

**Figure 3:**
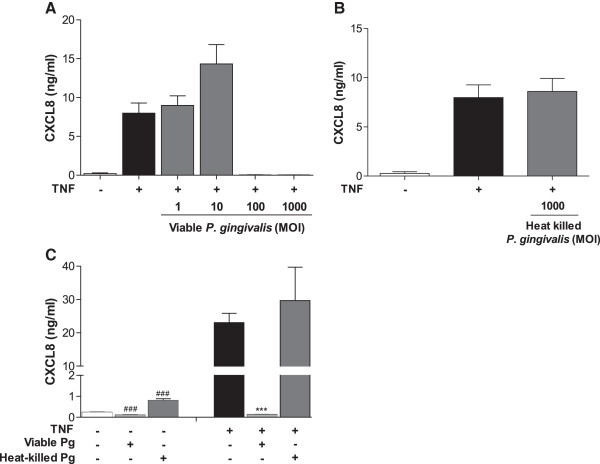
***P. gingivalis *is involved in the degradation of CXCL8 protein.** Dermal fibroblasts (50,000 cells/well) were stimulated with 50 ng/ml TNF-α for 6 h, where after the cells were treated with either the indicated concentrations of viable *P. gingivalis***(A)** or heat-killed *P. gingivalis* (MOI:1000) **(B)** for 24 h. CXCL8 levels were significantly suppressed by viable, but not heat-killed, *P. gingivalis*. **(C)** Gingival fibroblasts were stimulated with 50 ng/ml TNF-α for 6 h followed by treatment with viable or heat-killed *P. gingivalis* (MOI:100) for 24 h. Statistically significant differences compared to the negative control (#) or positive control TNF-α (*) were determined using Student’s *t*-test (###/***- p < 0.001).

We thereafter aimed to determine if the decreased levels of CXCL8, in response to viable *P. gingivalis*, were due to protein degradation. The fibroblasts were pre-treated with 50 ng/ml TNF-α for 6 hours to induce CXCL8 expression and accumulation. Thereafter, the fibroblasts were incubated with viable *P. gingivalis* (MOI:1, 10, 100 and 1000) or heat-killed *P. gingivalis* (MOI:1000) for 24 hours. The fibroblasts synthesized high levels of CXCL8 in response to TNF-α, which was further enhanced in the presence of viable *P. gingivalis* at MOI:10. However, higher concentrations of viable *P. gingivalis* (MOI:100 and MOI:1000), completely abolished the TNF-α-induced accumulation of CXCL8 (Figure 3A). In contrast, however, heat-killed *P. gingivalis* did not suppress TNF-α triggered CXCL8 levels (Figure 3B). These results were further confirmed by using gingival fibroblasts stimulated with viable and heat-killed *P. gingivalis*, with and without TNF-α pre-stimulation. CXCL8 basal levels were suppressed by viable *P. gingivalis* and by heat-killed *P. gingivalis* (Figure 3C). Furthermore, TNF-α-induced CXCL8 expression was suppressed below basal levels by viable bacteria, while heat-killed bacteria showed no alteration in the pre-accumulated CXCL8 levels.

### CXCL8 degradation is due to Arginine-gingipains

**Figure 4:**
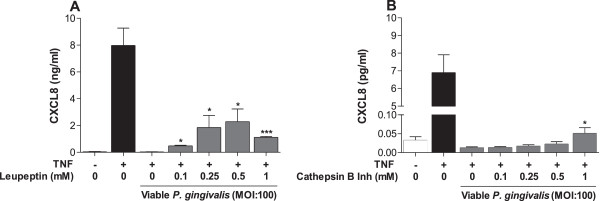
**CXCL8 degradation is due to Arginine-gingipains.** The involvement of Kgp and Rgp in CXCL8 degradation was determined by using Cathepsin B inhibitor II and Leupeptin. Viable *P. gingivalis* were incubated with the indicated concentration of inhibitor for 1 h prior to treatment of cells. Primary fibroblasts (50,000 cells/well) were stimulated with 50 ng/ml TNF-α for 6 h before the cells were incubated with *P. gingivalis* for 24 h. CXCL8 accumulation was more efficiently restored by Leupeptin **(A)** than with Cathepsin B inhibitor II **(B)**. The asterisks indicate significant differences compared to cells treated with *P. gingivalis*, without inhibitor. *- p < 0.05 ***- p < 0.001 (Student’s *t*-test).

To determine if *P. gingivalis* suppresses TNF-α induced CXCL8 release through Kgp and Rgp activities, viable *P. gingivalis* was incubated for 1 hour with increasing concentrations (0.1, 0.25, 0.5 and 1 mM) of cathepsin B II inhibitor or Leupeptin, before fibroblast infection. The fibroblasts were pre-stimulated with 50 ng/ml TNF-α for 6 hours and then incubated for 24 hours with treated or non-treated *P.gingivalis*. The Rgp inhibitor Leupeptin significantly reversed the *P. gingivalis*-induced suppression of CXCL8 at all concentrations (Figure 4A), whereas Cathepsin B II inhibitor at 1 mM only slightly changed the CXCL8 level (Figure 4B).

### *P. gingivalis* targets a wide range of fibroblast-derived inflammatory mediators

**Figure 5:**
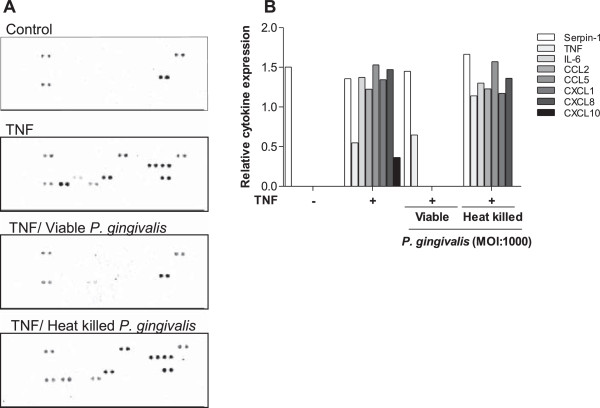
***P. gingivalis *targets a wide range of fibroblast-derived inflammatory mediators.** Fibroblasts (50,000 cells/well) were stimulated with 50 ng/ml TNF-α for 6 h before the cells were treated with viable, or heat-killed *P. gingivalis* (MOI:1000) for 24 h. The used cytokine array renders possible detection of the cytokines and chemokines specified in Table 1. Cytokine and chemokine levels were determined according to manufacturer’s instructions **(A)**. Treatment with viable *P. gingivalis* resulted in degradation of all inflammatory mediators except TNF-α and Serpin-1 **(B)**.

To examine if the immunomudulatory role of *P. gingivalis* accounts for inflammatory mediators other than CXCL8, a parallel determination of cytokines and chemokines was performed with a cytokine array (Table 1). Primary dermal fibroblasts (50,000 cells/well) were stimulated with 50 ng/ml TNF-α for 6 h before the cells were incubated with viable or heat-killed *P. gingivalis*, (MOI:1000), respectively (Figure 5). Non-stimulated fibroblasts were used as a control. TNF-α alone, or in combination with heat-killed *P. gingivalis,* induced secretion of TNF-α itself, as well as serpin-1, IL-6, CCL2, CCL5, CXCL1, CXCL10 and CXCL8. On the other hand, the levels of these inflammatory mediators, except TNF-α and serpine-1, were markedly suppressed by viable *P. gingivalis*. Heat-killed *P. gingivalis* did not change the TNF-α induced expression of the different inflammatory mediators, except an inhibition of CXCL10 and an enhancement (~ 2.5 fold) of TNF-α. The level of serpine-1 was consistently expressed at high levels independently of stimulation with TNF-α and/or bacteria.

## Discussion

The aim of the present study was to characterize the effects of *P. gingivalis* on human fibroblast inflammatory responses. The connection between periodontitis and atherosclerosis, as well as other systemic diseases, has suggested a role for periodontitis-induced bacteremia, including *P. gingivalis,* in stimulating and maintaining a chronic state of inflammation [2]. For instance, *P. gingivalis* DNA has been detected in atherosclerotic plaques [3,4] and in non-healing ulcers (unpublished data), however, to our knowledge, no previous studies on *P. gingivalis* infection of primary, human dermal fibroblasts have been performed. The fibroblasts are a source of connective tissue that maintain tissue haemostasis and integrity, and play an important role in tissue generation after wounding as well as in the pathogenesis of fibrotic inflammatory diseases and excessive scarring involving extracellular matrix accumulation [16]. Likewise, these cells have an active role in the innate immunity, although the immunity properties of fibroblasts have just begun to be revealed and many characteristics remain to be established [17,18]. In this study, we show that human skin fibroblasts, as well as human gingival fibroblasts, play an important part of the innate immune system by sensing microbial invasion and respond to it by producing and secreting inflammatory mediators, notably chemokines. Furthermore, we demonstrate that *P. gingivalis* has a direct modulatory function of the immune response of fibroblasts through the catalytic activities of gingipains targeting fibroblast-derived inflammatory mediators at the protein level.

Fluorescent micrographs showed that viable *P. gingivalis* adhered to and invaded dermal fibroblasts, suggesting that *P. gingivalis* utilizes strategies to evade the host immune response. This is in line with other studies that have shown *P. gingivalis* adhesion and invasion of oral epithelial cells, mainly mediated by gingipains and major fimbriae A. Invasion of epithelial cells, as well as gingival fibroblasts, is probably a mechanism applied by the bacteria to evade the host immune system and cause tissue damage, an important part of the pathogenesis of periodontitis [6,19,20]. For instance, this fimbriated strain of *P. gingivalis* has previously been shown to invade gingival epithelial cells after 90 minutes of incubation [21]. In this study we observed that *P. gingivalis* invaded dermal fibroblasts and had established an infection after six hours of incubation. In addition, after six hours of incubation was the CXCL8 level significantly reduced by *P. gingivalis*.

Consistent with previous observations [9,10], we show that short-term exposure of viable or heat-killed *P. gingivalis* (MOI:1000) induces CXCL8 production in fibroblasts. However, after 6 and 24 hours of incubation, viable *P. gingivalis* suppressed basal CXCL8 accumulation. On the contrary, heat-killed *P. gingivalis* increased CXCL8 levels, indicating that *P. gingivalis* possess heat-instable structures that are responsible for the degradation of CXCL8. In correlation, previous studies have shown that heat-killed *P. gingivalis* induces higher levels of inflammatory mediators, in particular IL-6 and CXCL8, than viable bacteria, suggesting degradation by the heat-instable gingipains [10,22]. To further investigate the effect of *P. gingivalis* on CXCL8, the fibroblasts were pre-stimulated with TNF-α, a well known inducer of inflammatory mediators. Lower doses of viable *P. gingivalis* (MOI:1 and MOI:10) in combination with TNF-α did not alter CXCL8 levels when compared to the positive TNF-α-stimulated control. However, higher concentrations (MOI:100 and MOI:1000) completely abolished the TNF-α-induced CXCL8 accumulation, while corresponding concentration of heat-killed *P. gingivalis* (MOI:1000) did not cause the same effects. This further implies that the suppression of CXCL8 is due to the proteolytic capacities of the gingipains. To test this theory and evaluate the importance of gingipains, we used cathepsin B inhibitor II and leupeptin, inhibitors of Kgp and Rgp, respectively. We found that *P. gingivalis*-mediated degradation is mainly dependent on Rgp. These findings are consistent with our previous findings, as well as results from others, showing that the gingipains from *P. gingivalis* degrades IL-2 and CXCL8, respectively [8,15]. However, inhibition of Rgp could only partially restore the CXCL8 levels, suggesting involvement of other proteolytic enzymes. It is also possible that a combination of Rgp and Kgp has a synergistic degradative effect, mediated by their specificity for cleavage after arginyl and lysyl residues, respectively. Furthermore, Dias and colleagues showed that there are two main types of CXCL8, a 72 amino acid variant, secreted by immune cells, and a 77 amino acid variant, secreted by non-immune cells. The latter was shown to have a lower chemotactic activity than the immune cell derived variant. However, upon cleavage by gingipains this shifted, and the 77 amino acid variant increased the chemotactic activity of neutrophils compared to the 72 amino acid variant [8]. Although gingipains degrade CXCL8, this consequently suggests that the chemotactic activity of CXCL8 increases upon stimulation of fibroblasts with *P. gingivalis*. However, more research is needed to determine the effects of *P. gingivalis*-derived proteolytic enzymes on the activity of these CXCL8 variants.

To investigate whether the gingipain-mediated effects of *P. gingivalis* also include other fibroblast-derived inflammatory mediators, we performed a relative cytokine assay which measured various cytokines and chemokines. This assay revealed that TNF-α stimulated primary, human skin fibroblasts produce CXCL8, TNF-α, IL-6, CCL2, CCL5, CXCL1 and CXCL10. Remarkably, the fibroblasts produced mostly chemokines, indicating that fibroblasts might play an important role as a link between the innate and the acquired immunity. All TNF-α induced inflammatory mediators, except TNF-α, were suppressed by viable *P. gingivalis*, strongly suggesting an effect of the gingipains *per se*. This shows that gingipains have a broad proteolytic capacity and targets a wide array of cytokines and chemokines, thereby interrupting several signaling pathways. The chemokines CCL2, CCL5, CXCL1 as well as CXCL10 are all important for recruiting immune cells to the site of infection, and by inhibiting their biological activity, *P. gingivalis* is able to modulate and diminish the level of infiltrating immune cells.

In contrast, viable *P. gingivalis* was not able to suppress TNF-α which is one of the most important inflammatory mediators. In fact, the level of TNF-α increased nearly two-fold by heat-killed bacteria, showing that *P. gingivalis* induce TNF-α expression in fibroblasts and, at the same time, degrade the TNF-α protein, although not extensively. Periodontitis is associated with a decreased abundance of fibroblasts [23] and TNF-α has been shown to be an important mediator of *P. gingivalis*-induced apoptosis. Graves *et al.* demonstrated that the numbers of apoptotic fibroblasts were significantly reduced in the absence of the TNF-receptor, suggesting that TNF-α-signalling is an important part in apoptosis of fibroblasts [24]. Thus, our results may indicate that *P. gingivalis* stimulates apoptosis of fibroblasts through a less extensive degradation of TNF-α and this could account for the fibroblast apoptosis that is a distinctive feature of periodontitis. Nevertheless, the degree of apoptotic fibroblasts after *P. gingivalis* infection need to be further investigated. In addition, it has been shown that the first nine residues of TNF-α N terminus are not needed for TNF-α protein to exhibit its biological activity [25]. Calkins and colleagues demonstrated that the two types of gingipains are able to individually degrade TNF-α, and also eliminate the biological activity [26].

CXCL10 is a chemokine with pleiotropic functions. It works as a chemoattractant for its CXCR3 (CXCL10 receptor) positive cells such as T cells, eosinophils, monocytes and NK cells, and it has also the capacity to induce apoptosis and regulate cell growth and proliferation, as well as angiogenesis [27,28]. Of interest, CXCL10 was the only chemokine that was suppressed by heat-killed as well as viable *P. gingivalis*, indicating that this chemokine is regulated by some additional mechanism beside that of heat-instable gingipains. For instance, a study by Ohno *et al.* showed that CXCL10 and CCL5 gene is induced by *P. gingivalis* in osteoblasts and ST2 mouse stromal cells and that NFκB inhibitor suppressed CCL5 expression but not CXCL10 [29]. This suggest that *P. gingivalis* modulates CXCL10 gene expression through an NFκB-independent pathway. Of further interest, the expression of CXCL10 is increased in autoimmunity diseases like rheumatoid arthritis and multiple sclerosis. For instance, Lee *et al.* reported that CXCL10 contributes to bone-resorption by inducing osteoclast formation in rheumatoid arthritis via induction of receptor activator of NFκB ligand (RANKL) [27]. However, since bone-resorption is a hallmark of progressive periodontitis, our results may indicate that CXCL10 plays a minor role when it comes to bone-resorption since even heat-killed *P. gingivalis* totally suppressed CXCL10. Besides, high levels of CXCL10 have receptor-independent anti-microbial properties. Even though it is questionable if such high levels (about 1 μM), *i.e.* concentrations 100-fold higher than needed for chemotactic function, are realistic *in vivo*, Prost and colleagues showed that this antimicrobial activity is achievable *in vitro* and may be an important response against bacterial infection [30]. Thus, the strong suppressive effect of CXCL10 by both viable and heat-killed *P. gingivalis* may in this case be beneficial for a sustained *P. gingivalis* infection. Anyway, further research is needed about the regulation of CXCL10 and its signaling pathways as well as its role in bacterial infection.

Serpin-1 (also known as plasminogen activator inhibitor 1), was continuously expressed regardless of stimulation with TNF-α and/or bacteria. Serpin-1 plays an integrated part of the plasmin system, working as an inhibitor of tissue plasminogen activator as well as urokinase plasminogen activator, both of which converts plasminogen to plasmin. Interestingly, serpin-1 has been implicated in fibroblast senescent. Serpin-1 is induced by various growth factors and has been suggested to be a down-stream target of p53, where p53 controls growth factor-dependent proliferation by upregulating serpin-1 [31]. However, the fibroblast strains in our experiments were used at low passages.

## Conclusion

In conclusion, our results show that a broad range of fibroblast-derived inflammatory mediators are inactivated by *P. gingivalis* due to proteolytic activities of gingipains, whereby the bacteria can create a more favourable milieu where it can evade the host immune system and promote its own growth and establishment. Also, by differentially regulate the inflammatory mediators, such as CXCL10 and TNF-α, *P. gingivalis* may have an impact on the composition of inflammatory cells infiltrate and the inflammatory process itself. Increased understanding of the role of fibroblasts in innate and acquired immunity and their interaction with periodontal bacteria is crucial for developing new strategies for preventing and treating periodontitis and related chronic inflammatory diseases.

## Competing interests

The authors declare that they have no competing interests.

## Authors’ contributions

HK, EP and TB designed the study. EP wrote the manuscript with HK and TB. EP and HK performed the experiments. All authors read and approved the final manuscript.
